# Association between habitual physical activity on episodic memory strategy use and memory controllability

**DOI:** 10.15171/hpp.2019.08

**Published:** 2019-01-23

**Authors:** Paul D. Loprinzi

**Affiliations:** ^1^Exercise & Memory Laboratory, Department of Health, Exercise Science and Recreation Management, The University of Mississippi, University, MS 38677, USA

**Keywords:** Episodic memory, Exercise, Movement, Perceptions, Self-efficacy

## Abstract

**Background: ** The primary objective of this study was to evaluate the association between habitual physical activity engagement and perceived controllability of memory function. Secondary objectives included the evaluation of physical activity on memory strategy use, and whether the latter mediates the relationship between physical activity on memory controllability.

**Methods:** Two-hundred and nine young adults (M_age_=25 y) completed a brief survey evaluating physical activity (Physical Activity Vital Signs Questionnaire), memory strategy use (Memory Functioning Questionnaire), and memory controllability (Memory Controllability Inventory).

**Results: ** Physical activity was not associated with memory strategy use (β=0.68; 95% CI: -1.25,2.62; P=0.48), nor was memory strategy use associated with memory controllability. Physical activity was consistently associated with various attributes of memory controllability, including Present Ability (β=1.10; 95% CI: 0.07, 2.12; P=0.03), Potential Improvement (β=0.84; 95% CI:0.05, 1.63; P=0.03), Effort Utility (β=0.87; 95% CI: 0.11, 1.61; P=0.02), Inevitable Decrement (β=-1.19; 95% CI: -2.19, -0.19; P=0.02) and Alzheimer’s likelihood (β=-1.21; 95% CI: -2.29,-0.12; P=0.02).

**Conclusion: ** Physical activity is consistently associated with greater perceptions of memory controllability. Future longitudinal and experimental work on this topic is warranted to evaluate if physical activity can foster an individual’s ability to modify their behavior and cognitions to enhance and preserve memory function.

## Introduction

The field of memory function is not new, but the potential role of physical activity in subserving memory function is an emerging line of inquiry. Various laboratories,^1-5^ including ours,^6-14^ has demonstrated that both acute and chronic engagement in physical activity may enhance memory function. From an acute exercise perspective, exercise may help to facilitate the recall of memories within a spatiotemporal context (episodic memory) from alterations in neuronal excitability, and ultimately, modulation of long-term potentiation and synaptic plasticity.^10^ Further, chronic exercise engagement may subserve memory function via the widespread adaptations from exercise, including neurogenesis, gliogenesis, and angiogenesis.^10,15^

In addition to the importance of monitoring and evaluating objective memory performance, it is also important to evaluate subjective memory outcomes (e.g., complaints made by individuals about their cognitive symptoms), as, for example, subjective memory complaints have been shown to predict negative memory outcomes.^16,17^ Even among young adult populations, subjective memory complaints are prevalent,^18^ and this population has also been identified as a point in time when objective memory function may start to decline.^19^

Individuals, even young adults, may employ various techniques to try and control, improve, or preserve their memory function. Among others, one cognitive technique may be the utilization of mnemonic strategies, which often includes techniques that involve organization, elaboration, and visual imagery (e.g., method of loci). Although experimental data suggests that acute and chronic physical activity may help to improve memory performance on objective assessments, we have a limited understanding of whether habitual physical activity engagement is associated with an individual’s perception of memory controllability. I hypothesize that higher levels of physical activity will be associated with greater perceptions of memory controllability (e.g., increased perception that their memory can be improved as well as preserved during aging). This hypothesis is plausible as prior work has shown that people believe that physical activity may be an effective behavior to help treat and prevent various cardiovascular and cognitive outcomes.^20^ I also hypothesize that this potential relationship between physical activity and memory controllability may be influenced by the utilization of memory-based strategies, such as mnemonics. That is, higher levels of physical activity may be associated with greater memory controllability via memory strategy utilization. Such an effect is plausible as other work demonstrates that memory strategy use may play an important role in memory performance among healthy individuals.^21^

Couched within the above, the primary objective of this study was to evaluate the association between habitual physical activity engagement and perceived controllability of memory function. Secondary objectives included the evaluation of physical activity on memory strategy use, and whether the latter mediates the relationship between physical activity on memory controllability.

## Materials and Methods

### Study design and participants

A random sample of 1500 college students at the University of Mississippi were sent an e-mail inviting them to participate in a brief on-line survey. Thus, all variables were assessed via this on-line survey. This study was approved by the University of Mississippi’s institutional review board. Completion of the survey provided voluntary participant consent. A total of 245 students completed the survey, and those who self-reported being pregnant, having a concussion in the past 30 days or having a diagnosis of a cognitive impairment, were excluded from the analyses. In total, 209 eligible participants provided complete data for all variables (list-wise deletion analysis used for missing data), constituting the analytic sample.

### Exercise behavior

Subjective assessment of exercise was assessed using the two-item (days per week and minutes per day) physical activity vital signs (PAVS) questionnaire, indicating the number of minutes per week engaged in moderate-to-vigorous physical activity (MVPA). Meeting physical activity guidelines was defined as at least 150 min/wk of MVPA. This assessment has demonstrated evidence of validity.^22-26^ Notably, this self-report MVPA measure correlates with accelerometer-assessed MVPA (r = 0.52, *P*<0.001).^23^

### Memory strategy use

Participants completed mnemonic usage questions from the Memory Functioning Questionnaire.^27^ Specifically, 8 questions were employed. Participants were asked “*how often do you use these techniques to remind yourself about things*?” The different techniques included: keep an appointment book; write yourself reminder notes; make lists of things to do; make grocery lists; plan your daily schedule in advance; mental repetition; associations with other things; and keep things you need to do in a prominent place where you will notice them. For each of these 8 potential strategies, options ranged from 1 (always) to 7 (never). In the present sample, internal consistency for these 8 strategies, as measured by Cronbach’s alpha, was 0.82.

### Memory controllability

Memory controllability was evaluated using the Memory Controllability Inventory,^28^ consisting of 19-items. This instrument includes 6 subscales, including Present Ability (3 items), Potential Improvement (3 items), Effort Utility (3 items), Inevitable Decrement (3 items), Independence (3 items), and Alzheimer’s Likelihood (4 items). An example item for Present Ability is, “*I can remember the things I need to*.” An example item for Potential Improvement is, “*I can find ways to improve my memory*.” An example item for Effort Utility is, “*If I work at it, I can improve my memory*.” An example item for Inevitable Decrement is, “*There’s not much I can do to keep my memory from going downhill*.” An example item for Independence is, “*As I get older, I won’t have to rely on others to remember things for me*.” Lastly, for Alzheimer’s likelihood, an example item is, “*I think there’s a good chance I will get Alzheimer’s disease*.” For all questions, response options ranged from 1 (strongly disagree) to 7 (strongly agree).

For each subscale, responses were summed for the respective items. For Present Ability, higher scores indicate a greater perceived present ability in memory. For Potential Improvement, higher scores indicate a greater perceived ability to improve their memory. For Effort Utility, higher scores indicate a greater perceived effort can help facilitate memory. For Inevitable Decrement, higher scores indicate that a decrement in memory is inevitable (i.e., not favorable). For Independence, higher scores indicate a greater perceived independence in their memory as they age. Lastly, for Alzheimer’s Likelihood, higher scores indicate that the development of Alzheimer’s disease is likely to happen to them (i.e., not favorable). For the present sample, internal consistency for this entire memory controllability inventory was 0.85.

### Covariates

Self-reported covariates included age, gender, race-ethnicity, body mass index (from self-reported height and weight), and self-reported smoking status (current, former, never). These parameters were selected given their potential association with memory function.

### Statistical analyses

All statistical analyses were computed in Stata (v. 12, College Station, TX, USA). Multivariable linear regression models were computed that examined the association between exercise and memory self-efficacy. Models were computed separately for memory strategy use, and similarly, models were computed separately for each of the subscales of the memory controllability inventory. In all models, covariates included age, gender, race-ethnicity, body mass index, and self-reported smoking status. Statistical significance was set at α = 0.05.

## Results

Table 1 displays the characteristics of the sample. The mean (SD) age of the participants was 25 (8.8) years old, with the majority being female (64%) and non-Hispanic white (75%). Forty-two percent of the sample met physical activity guidelines.

Table 2 displays the multivariable regression results examining the association between physical activity on memory strategy use and memory controllability. Results are displayed for those meeting (vs. not) physical activity guidelines, as when physical activity was expressed as a continuous variable, results were similar (results not shown). Physical activity was not statistically significantly associated with memory strategy use when memory strategy use was expressed as a sum of the responses from the 8 memory strategy variables (β = 0.68; 95% CI: -1.25, 2.62; *P* = 0.48). Notably, physical activity remained non-significantly associated with memory strategy use when results were evaluated for each separate memory strategy (results not shown).

Also shown in Table 2 are the results examining the association between physical activity and memory controllability. Physical activity was consistently associated with memory controllability (5 out of the 6 domains). Those who met physical activity guidelines (vs. not) had statistically significantly *higher* perceptions of Present Ability (β = 1.10; 95% CI: -0.07, 2.12; *P* = 0.03), Potential Improvement (β = 0.84; 95% CI: 0.05, 1.63; *P* = 0.03) and Effort Utility (β = 0.87; 95% CI: 0.11, 1.61; *P* = 0.02), and also *lower* perceptions of Inevitable Decrement (β = -1.19; 95% CI: -2.19, -0.19; *P* = 0.02) and Alzheimer’s Likelihood (β = -1.21; 95% CI: -2.29, -0.12; *P* = 0.02). Notably, additional models were evaluated that included memory strategy utilization as an independent variable, and for all models, memory strategy utilization was not associated (*P*’s>0.05) with any of the memory controllability measures (results not shown).

## Discussion

The primary purpose of this study was to evaluate if, among a young adult sample, habitual engagement in physical activity was associated with memory controllability, and whether this potential association was influenced by memory strategy utilization. The motivation for this evaluation stemmed from previous work highlighting that both acute and chronic physical activity have been shown to improve objective measures of memory,^29^ as well as other work suggesting that young adults believe that physical activity may be a useful behavior to help improve cognitive outcomes.^20^ The main findings of this study were: (1) Physical activity was not associated with memory strategy utilization, (2) Memory strategy utilization was not associated with memory controllability, but (3) Physical activity was consistently associated with memory controllability.

Our null findings regarding memory strategy use may be a result of the limited number of memory strategies that were assessed. Perhaps a more comprehensive evaluation of memory strategy use would have increased the likelihood of observing a relationship between memory strategy use with physical activity and/or memory controllability. Although we restricted our memory strategy evaluation to commonly used mnemonic techniques, there are various learning strategies across multiple levels of cognitive processing (e.g., chaining/chunking, spatial clustering, affective clustering, overt rehearsal techniques, schema learning). However, we focused on commonly used mnemonic techniques, as these types of strategies may help to subserve memory function.^30-36^ Future research is needed to determine if indeed physical activity plays any role in influencing the utilization of memory strategies. Such a relationship is theoretically feasible. For example, executive functioning, known to be influenced by exercise,^37^ may also influence memory strategy use.^38^

A notable observation of this study was that, among this young adult population, habitual engagement in physical activity was favorably associated memory controllability. Although we did not objectively measure memory function, our observation of an association between physical activity and memory controllability could have been a result of a higher memory controllability effect among active individuals because of their potential greater memory function. However, not all studies demonstrate a memory enhancing effect from exercise,^39^ and thus, there may be other possible explanations for our observations. One potential explanation is that, in theory, physical activity may foster an enhanced self-efficacy, or perceived controllability, in other domains. This transference paradigm (global self-efficacy) has been discussed in other behavioral domains, in that, physical activity is thought to enhance one’s self-efficacy to change other behaviors (e.g., diet and smoking) via improvements in executive functioning.^40-42^ In the context of the present study, and although speculative, perhaps physically active individuals have enhanced global self-efficacy, and as a result, feel confident in modifying their lifestyle in a way that would be conducive to enhance and preserve their memory function. Such a transference paradigm is interesting and worthy of future exploration, as its implications have large individual and societal ramifications.

In conclusion, the purpose of this study was to evaluate the interrelationships between habitual physical activity, memory strategy use, and memory controllability. The main findings of this study suggest that physical activity is consistently associated with greater perceptions of memory controllability. Future work should overcome the limitations of this study (e.g., cross-sectional design, self-report measure of physical activity, limited study population). For example, longitudinal and experimental work on this topic is warranted to evaluate if, indeed, objectively-measured physical activity can foster an individual’s ability to modify their behavior and cognitions to enhance and preserve memory function.

## Ethical approval

This study was approved by the University of Mississippi’s ethics committee (Protocol #18x-300).

## Competing interests

The author declares no competing interest.

## Funding

None.

## Author’s contribution

PDL conceived the study, collected the data and wrote the entire manuscript.

**Table 1:** Characteristics of the study sample (N = 209)

**Variable**	**Point Estimate**	**SD**
Age, mean years	25.4	8.8
Gender, % female	64.5	
Race-Ethnicity, % White	75.1	
Body mass index, mean kg/m^2^	25.6	6.2
Current smoker, %	3.8	
MVPA, mean min/week	159.1	155.4
Meeting physical activity guidelines, %	42.1	
Strategy score, mean overall	22.7	6.9
Present ability, mean	15.9	3.5
Potential improvement, mean	16.2	2.7
Effort utility, mean	16.2	2.6
Independence, mean	13.8	3.1
Inevitable decrement, mean	10.0	3.4
Alzheimer’s likelihood, mean	12.3	3.8

MVPA, Moderate-to-vigorous physical activity. Meeting physical activity guidelines defined as at least 150 min/week of MVPA.

**Table 2:** Multivariable regression results examining the association between physical activity on memory strategy use and memory controllability (N=209)

**Model**	**Unstandardized Beta Coefficient**	**95% CI**	***P*** ** value**
**Memory Strategy Use**			
Meeting MVPA guidelines vs. not	0.68	-1.25, 2.62	0.48
**Memory Controllability**			
**Present Ability**			
Meeting MVPA guidelines vs. not	1.10	0.07, 2.12	0.03
**Potential Improvement**			
Meeting MVPA guidelines vs. not	0.84	0.05, 1.63	0.03
**Effort Utility**			
Meeting MVPA guidelines vs. not	0.87	0.11, 1.61	0.02
**Inevitable Decrement**			
Meeting MVPA guidelines vs. not	-1.19	-2.19, -0.19	0.02
**Independence**			
Meeting MVPA guidelines vs. not	0.58	-0.31, 1.48	0.20
**Alzheimer’s likelihood**			
Meeting MVPA guidelines vs. not	-1.21	-2.29, -0.12	0.02

MVPA, Moderate-to-vigorous physical activity. Meeting physical activity guidelines defined as at least 150 min/wk of MVPA. Each model controlled for age, gender, race-ethnicity, body mass index, and smoking status.
